# Canadian public health experiences during COVID-19: a new framework for assessing evidence

**DOI:** 10.3389/fpubh.2025.1620514

**Published:** 2025-09-23

**Authors:** Christopher S. Cotton, Monica C. LaBarge, Ardyn Nordstrom

**Affiliations:** ^1^Department of Economics and School of Medicine, Queen’s University, Kingston, ON, Canada; ^2^Queen’s University, Kingston, ON, Canada; ^3^School of Public Policy and Administration, Carleton University, Ottawa, ON, Canada

**Keywords:** COVID-19, evidence-based policy, evidence hierarchy, public health decision-making, crisis, uncertainty, Canada

## Abstract

**Background:**

Public health emergencies like COVID-19 require public policy and practice decisions at a time of uncertainty and rapidly changing science.

**Methods:**

We conducted qualitative, phenomenological interviews with 25 senior Canadian public health leaders at local, provincial, and federal levels. Interviews explored how evidence was assessed, interpreted, and utilized during Canada’s COVID-19 pandemic response. Data analysis followed rigorous inductive coding to identify key themes.

**Results:**

Participants highlighted limitations in traditional evidence hierarchies, emphasizing instead the critical role of timely, context-specific information such as predictive modeling, local surveillance data, and stakeholder insights. Officials described dynamically balancing methodological rigor with evidence credibility and applicability. We propose the Methodology-Credibility-Applicability (MCA) Evidence Framework, emphasizing simultaneous assessment across these three dimensions.

**Discussion:**

We document the experiences of public health leaders during the COVID-19 crisis, focusing on the assessment and use of evidence in decision making. The results challenge established hierarchies for assessing evidence and highlight the need for flexible, multidimensional frameworks for evaluating evidence during crises.

## 1 Introduction

The COVID-19 pandemic required rapid public health decisions under substantial uncertainty, incomplete scientific understanding, and rapidly evolving evidence. Public health decision-makers frequently needed to act without insights from the types of studies traditionally considered essential for evidence-based policy, such as randomized controlled trials or systematic reviews [e.g., (1, 2)]. Instead, officials needed to rapidly synthesize, interpret, and apply a diverse range of evidence, including international observational studies, predictive models, preprints, expert opinion, anecdotal accounts, and community-generated data, while simultaneously managing public expectations, economic impacts, and political pressures [e.g., (3)].

In Canada, the complexity of the pandemic response was amplified by the multilevel governance structure of public health, with roles and responsibilities delineated across federal, provincial/territorial, and local health authorities. This structural complexity required officials not only to evaluate evidence from diverse sources but also to interpret and apply it within varied local contexts (4). Although the literature on evidence-based policy (EBP) in public health extensively addresses methodological quality assessment [i.e., the traditional “evidence hierarchy,” (2)], it provides limited guidance on systematically evaluating the credibility of evidence sources and the local applicability of research findings, particularly under crisis conditions.

In this study, we provide insights into how Canadian public health leaders navigated these complexities during the COVID-19 pandemic, based on detailed qualitative interviews with 25 senior decision-makers from across federal, provincial, and local jurisdictions. Drawing on theoretical frameworks from crisis decision-making (3), EBP, and the sociology of scientific knowledge (SSK) (5, 6), our analysis provides insights into the types of evidence used in decisions, and how evidence quality, source credibility, and contextual relevance were dynamically assessed over the course of the pandemic. Our empirical findings highlight the pragmatic and multidimensional nature of evidence assessment during crises, challenging traditional evidence hierarchies and pointing toward the need for more flexible frameworks.

We introduce the Methodology-Credibility-Applicability (MCA) Evidence Framework to systematically capture the multidimensional approach to evidence assessment used by public health decision-makers during crises. Unlike traditional evidence hierarchies that prioritize methodological rigor alone, this new conceptual MCA framework emphasizes simultaneous evaluation across these three key dimensions. Our analysis demonstrates that during public health emergencies, timely and context-specific evidence such as localized surveillance data and predictive models, often holds greater practical value than more robust but less applicable international research. During crises, public health officials lack systematic standards for assessing evidence credibility and applicability, highlighting a critical gap in evidence-based practice that the MCA Framework directly addresses.

This paper thus contributes to the broader literature on evidence-informed decision-making in public health by proposing a structured yet flexible approach suitable for crisis contexts. In doing so, it identifies implications for policy and practice, emphasizing the need to strengthen infrastructure for rapid evidence synthesis, improve transparency and interpretability of predictive modeling, and establish formalized criteria for credibility and applicability assessments. These recommendations aim to enhance public health preparedness, improve interdisciplinary collaboration, and support more effective communication of scientific uncertainty in future public health emergencies.

## 2 Background and methodology

This study used a qualitative research design with a phenomenological approach (7), collecting data via interviews to examine the lived experience of public health decision-makers in Canada during the COVID-19 pandemic. In these interviews, we documented public health experts’ perceptions of the role and challenges of evidence use within their organization and in guiding policy during the pandemic.

### 2.1 The Canadian context

Canada’s public health system is decentralized, dividing responsibilities among federal, provincial/territorial, and local governments. The Public Health Agency of Canada (PHAC) provides national guidance, manages borders, and coordinates vaccine procurement. Provincial and territorial governments, led by Chief Medical Officers of Health (CMOHs) or their equivalent, oversee healthcare delivery and major public health measures, while local Medical Officers of Health (MOHs) adapt these guidelines to regional conditions, manage case and contact tracing, and implement community-specific interventions (31, 32). This multi-layered system created significant regional variation in Canada’s pandemic response, influenced by diverse geographic, demographic, and healthcare capacity factors (33).

Despite these specific institutional features, Canada’s COVID-19 experience shares key similarities with other decentralized, democratic countries such as Australia, Germany, and the United States. Common challenges include balancing national consistency with local flexibility, managing rapid evidence evolution, and navigating political and social pressures in public health decision-making (34). Thus, while aspects of Canada’s experience are unique, insights gained from Canadian public health officials likely hold broader relevance for understanding evidence-informed decision-making in public health emergencies internationally.

### 2.2 Interview participants and procedures

A purposive sample of 25 participants was recruited to capture a diverse range of perspectives from individuals involved in public health decision-making across Canada. The sample included nine Chief, Deputy Chief, and Associate Medical Officers of Health (or equivalent roles) at provincial, territorial, and regional levels, including current or former heads of several provincial and territorial public health agencies, five senior public health officials within the federal government, nine advisors to Medical Officers of Health (or equivalent roles) within public health agencies or units, and two senior advisors to public health agencies working outside of traditional public health agencies or units. Several participants held secondary affiliations with academic institutions. Table 1 provides examples of the types of roles participants had, and the jurisdiction level they operated in.

**Table 1:** Participant details.

Jurisdictional level (anonymity label)	Number of participants	Representative titles
Local (L)	5	Associate Medical Officers of Health and COVID-19 Outbreak Team Managers, Advisors to Medical Officer of Health
Provincial/territorial (PT)	13	Chief Medical Officers of Health (or equivalent), Deputy Chief Medical Officers of Health (or equivalent), Executive Directors of Health Systems
National (N)	7	Scientific Directors, Managers of Policy Research, Executive Directors

Due to the prominence of the roles of our participants and concerns about identifiability, we have masked references to jurisdictions or actions to preserve anonymity, using L, PT, and N to refer to individuals in local, provincial/territorial, or national jurisdictions, respectively. The sample included individuals with experience in most provinces and territories, providing a broad representation of the Canadian public health landscape.

To recruit interview participants, an initial call for interview participants was distributed to the mailing list for Chief and Associate Medical Officers of Health across Canada. Additional participants were recruited through an open call distributed to the members of the One Society Network, a network of health researchers across North America. Recipients either self-selected to be interviewed or recommended the research team contact other individuals. This approach led to the inclusion of stakeholders from a wide range of regions, public health jurisdictions, and seniority levels.

As participants were interviewed, we used a snowball sampling technique to identify additional interview participants through direct referrals. As participants mentioned specific organizations or stakeholders within the public health system that would be relevant to speak to, the research team reached out to the relevant officials within these organizations to request an interview, maintaining the anonymity of the individuals who made the reference if they wished to be kept anonymous. By using this snowball sampling approach, the research team specifically asked for referrals to jurisdictions that were underrepresented by participants to date, aiming to increase the representativeness of participants across geographic regions, jurisdictions and roles within public health organizations. Additional interviews were solicited until saturation was met across major themes. This produced a sample that included viewpoints from multiple perspectives within public health organizations, as well as across jurisdictional levels (local, provincial/territorial, national) and most geographic regions.

Between December 2022 and July 2023, two of the authors (ML and AN) jointly conducted in-depth interviews using a semi-structured interview guide. The interview guide focused on participants’ decision-making and advisory roles over the course of the pandemic, the types and sources of evidence used or considered, and how they perceived political, economic, and social factors as influencing decisions related to public health. During these interviews, participants were asked about how the COVID-19 pandemic influenced each of these areas.

Interviews were conducted virtually via Zoom, providing flexibility for participants across Canada. The interviews ranged from approximately 30 to 75 min. If the participant provided consent to have the interview recorded, the interviews were audio-recorded and transcribed verbatim, resulting in 390 pages of transcribed data. One participant responded to the interview questions in via email. All transcripts were anonymized during the transcription process.

### 2.3 Data analysis

Consistent with our phenomenological orientation, we sought to capture and interpret participants’ experience and perceptions related to evidence use in public health. We utilized a three-stage process (8):

1. *Reading for key ideas*: Each interviewer (ML and AN) read all transcripts in-depth to identify preliminary concepts and recurring motifs.
2. *Theme emergence*: Through repeated reading and note-taking, emerging themes were identified across interviews. The thematic analysis was supported with the use of Dedoose 9.0, which allows multiple team members to apply themes and iteratively update the codebook as themes emerge from the data.
3. *Elevation to theoretical contributions*: Themes were scrutinized for broader patterns and connections to existing literature in public health decision-making.

All transcripts were hand-coded using Dedoose 9.0, beginning with a set of initial codes drawn from the interview guide (e.g., “evidence sources used in public health decisions,” “priorities in public health,” “processes for making decisions”). As analysis progressed, new codes were added or refined to reflect novel concepts, and were aggregated to sub-themes as connections between them began to emerge. Both coders (ML and AN) worked independently to review and annotate transcripts, then convened regularly to compare interpretations, resolve discrepancies, and revise the codebook as needed. Through these discussions, the authors identified emergent themes within the data. Consistent with Castleberry and Nolen (9) employing multiple coders, and regular iterations to refine the codebook ensured the emergent themes were replicable across coders. The emergent themes were further reviewed by the third team member (CC) to further confirm the emergent codes in the data. Finally, those emergent themes and the broader perspective on evidence they represented were elevated to inform the development of the MCA framework.

### 2.4 Ethics

The study received ethical approval from Queen’s University (Approval: 6037505) and Carleton University (Protocol: 118724) prior to data collection. All participants who participated in an interview provided informed consent to participate in the study, including consent to audio-record and transcribe the interviews. One additional participant consented to participating through written responses. All participants were assured of the confidentiality and anonymity of their responses.

## 3 Results

This study focuses on how, during COVID-19, decision-makers navigated incorporating evidence from a wide range of sources into decision- and policymaking, how the use of different sources of evidence evolved over the pandemic, and how decision-makers balanced scientific reasoning with social, economic, and political factors. The findings presented here are derived from the themes that emerged from interviews transcripts regarding how public health experts judged and used evidence while making public health decisions. Several key themes were identified related to the use of first principles, how the quality of different evidence sources was assessed, and how these evidence sources were contextualized with local evidence sources. Together, these themes inform the development a new framework for evidence assessment, presented in Section 4.

### 3.1 Early pandemic: reliance on first principles and lower-tier evidence

In the context of early COVID-19, when public health leaders were faced with an unknown virus and limited research, they relied largely on “first principles”—established scientific knowledge about core public health practices and managing communicable diseases. This type of information is foundational in public health education and practice, and familiar to public health officials. Several participants across multiple jurisdictions indicated that epidemiological principles and other lessons learned from previous respiratory virus outbreaks informed policies even before COVID-19 evidence emerged:

*“There were not a lot of things being published at the beginning. So, in many cases, it was going back to core public health principles [and] evidence that we knew for other interventions and other conditions”—N6.*

Most practitioners interviewed for the study referred to these kinds of first principles as essential for informing decisions and recommendations, such as isolation and quarantine recommendations to manage outbreaks of highly communicable infectious disease, particularly early on during the pandemic. Indeed, like other contexts (10), Canada’s public health institutions have evolved to respond to this kind of emergency by leveraging the experience practitioners have developed through past crises. In 2003, for example, Canada managed an outbreak of the highly infectious severe acute respiratory syndrome (SARS), which prompted a revision of public health policies and systems (4). Even once COVID-19 began to spread, established scientific research on COVID-19 was scarce. Participants consistently described using “lower-tier evidence” despite its place at the bottom of traditional hierarchies [e.g., (1, 2)], due to the need to understand emerging threats and estimate impacts. Given the urgency, pre-prints were necessary, but as one participant noted, they contributed to an “uncertain landscape” (L4) around how the evidence used might change. Many others echoed this sentiment, noting that this uncertainty required additional screening. This aligns with concerns about misinformation and the need for caution when interpreting unreviewed research (11). Although some evidence suggests that during COVID, data from these kinds of sources were retracted at higher rates (12), they still provided guidance when combined with contextual knowledge, first principles, and transparent discussions of limitations. One participant noted:

*“I went back and looked at to what extent the pre-prints were altered when they ended up peer-reviewed. [.] The answer was infrequently”*—*N1.*

This was echoed by others, who noted that all evidence, not just preprints, is subject to scrutiny when evaluating the quality and relevance of evidence. Beyond preprints, case studies and evidence from international public health agencies like the CDC, WHO, and NHS, particularly from early affected countries like China and Italy, were key sources of early evidence. In the absence of higher-level evidence on the evidence hierarchy, multiple decision makers across all levels of public health agencies described how such sources were deemed valuable in the uncertain and urgent environment, despite potential limitations of applying findings from different contexts, as illustrated by the following quote:

*“Jurisdictional scans are a part of that decision-making process. So, when decisions are being made, one of the pieces of evidence being presented is what’s happening around the world, because it is important and it obviously does inform what we do”—N3.*

Even when evidence existed, several participants noted the challenges associated with extrapolating evidence from one context to another. This was most evident when participants were describing the process of extrapolating insights when there were regional differences, with participants in all regions and at all levels noting that it was not appropriate to automatically assume evidence from one region was relevant in another. This is illustrated by the following quote:

*“I would not want to be making some assumptions about how it would work in Atlantic, Canada, when I do not live there and never have lived there and do not really know how it would work”—N7.*

However, many PH leaders saw evidence from other regions and public health settings as valuable information when there was limited local data or standardized guidance. Although they considered such evidence in their decision-making, it was widely acknowledged that such data was less conclusive and could be interpreted in a variety of ways, even by experts. This was a common theme described by subjects at in local, provincial/territorial, and national levels:

*“Different Medical Officers of Health are going to integrate evidence in different ways, based on their values and expertise”—L2.*

*“Even with the same data, different people will make different decisions based on their values, their beliefs, and their experience. I think that’s a huge player in decision making”—PT1.*

*“If you show the same data set to 10 people, they’ll interpret it in 10 different ways”—N3.*

Beyond biomedical evidence, multiple subjects, particularly at the local level, noted the lack of evidence surrounding best practices for the *implementation* of public health activities and interventions in the early days of the pandemic. This is illustrated in the following quote from one local official:

*“There is very little literature on operations. [.] There are all these different forms of how you set up a vaccine clinic. Which one’s best? I still do not know. […] There’s none of that out there, […] even things like case and contact management protocols”—L3.*

These descriptions of evidence sources used at the onset of the COVID-19 public health emergency highlight the role of expert judgment in bridging the gap between limited evidence and the need for practical action, a common theme in crisis decision-making when established protocols may be insufficient (13).

### 3.2 The rise of local evidence, predictive models, and evidence synthesis

After the information-scarce early days, decision-making shifted to rely more heavily on local data, predictive models, and peer-reviewed evidence syntheses. This reflects the changes in the evidence landscape during a crisis, moving from reliance on general principles and external data to more context-specific evidence and sophisticated analytical tools.

#### 3.2.1 Emphasis on local data and applicability of evidence

Within local public health units across the country, participants described a shift toward prioritizing local data as it emerged. One participant noted:

*“At the beginning of the pandemic, […] we were weighing global experience more heavily because we should learn from what they were experiencing. However, now, as time has gone on, we weigh local evidence more. We barely look at global evidence”*—*PT7*.

This shift reflects the need to adapt interventions to local conditions, a principle of effective evidence-based public health policy and practice (35, 36). Others noted that the way evidence was integrated and the emphasis that was put on different levels of evidence evolved as the pandemic progressed. Decision makers across all levels of public health reported that evidence at the provincial and local levels increasingly drove decisions as the pandemic evolved. This reflects the oft-cited axiom that “public health is local” (14), and that local evidence is key to crafting appropriate decisions and policies. This need for locally relevant evidence was among the most widespread and consistent themes that emerged from the data.

#### 3.2.2 Predictive models and new local evidence sources

Epidemiological models played a central role in projecting outcomes under different scenarios and informing decisions about public health measures. National epidemiological modeling work done by the Public Health Agency of Canada was widely commended as an excellent source of information incorporating feedback from sub-national public health units:

*“[The PHAC national modeling effort] was excellent. [.] They heard our criticisms, fears, worries, and hopes for this, and I could see it evolve with that input. So, in my opinion, they did a very, very good job of two-way communication around modeling. That was kind of helpful. But it’s a country perspective; not so much for a province or territory”—PT7.*

This again highlights the tension between local and national evidence sources, as local and provincial public health units were tasked with operationalizing evidence that often came from more general populations. Although the specific tension between national and subnational jurisdictions may be specific to the Canadian public health context, multiple experts emphasized the importance of adopting an iterative process to develop models that reflect the dynamic nature of modeling, with models being updated and refined as new data became available and the understanding of the virus evolved. This included the development of new data and evidence sources, such as wastewater treatment data, which often resulted from new pandemic-driven collaborations with local partners, such as universities and utility companies.

*“With wastewater surveillance, every public health unit is in this type of tripartite relationship where the academics lead the analysis…”—L3.*

While others at provincial levels noted that “*just now, we are actually really understanding across the country the value of wastewater surveillance*” (PT11), several individuals noted that integrating this new kind of evidence presented its own initial challenges. Initially, decision-makers in multiple regions and across all jurisdictions lacked experience with this kind of evidence; though, it evolved to become a common input into evidence-based decision-making, providing applicable local evidence:

*“Wastewater information and the research initiatives that started that up with great enthusiasm made good headway. But, then it was not really an integrated part of surveillance and wasn’t terribly valuable at the beginning for direct management of COVID-related issues. But over time, you know that there became a pathway for it now to be included and incorporated with clinical information”—PT12.*

More broadly, participants acknowledged the challenges associated with the use of disease-predictive models, including lack of capacity, with one participant noting these difficulties were not experienced equally across the country. These quotes are illustrative of broader themes related to experts’ understanding of the models’ limitations and the challenges associated with turning results from epidemiological models into policy decisions [e.g., (2)].

#### 3.2.3 Credible evidence synthesis

Organizations like PHAC, provincial public health authorities, and provincial science tables helped assess and synthesize the growing body of evidence, including model outputs, and providing recommendations to decision-makers. One participant described the value of these organizations:

*“Within a couple of months the volume of new publications was so huge, one person could not necessarily read through all of that, especially not while trying to do all the other things. So, then I really relied on synthesis groups”—PT9.*

The expressed desire for credible, high-capacity knowledge synthesis systems to support public health decisions across was present across regions and government levels in our data. While most participants emphasized the value of evidence syntheses from the PHAC-coordinated Scientific Advisory Committee, this was particularly valuable in smaller jurisdictions, which had less capacity to conduct these kinds of syntheses.

Although the need for credible syntheses is likely universal across contexts, there are some insights that may be particularly relevant for the Canadian context, with its federalist government system and fairly high level of independence across provinces. Some participants noted duplication of effort across jurisdictions, with provinces such as British Columbia and Quebec developing their own synthesis and advisory structures rather than relying on federal outputs. This highlights both the value and fragmentation of Canada’s evidence ecosystem during the pandemic, with multiple participants emphasizing the need for more responsive review structures:

*“There were times when the same review was being done by multiple groups, because it was just so hard to keep track of who all was doing what”—N7.*

Furthermore, several participants highlighted capacity constraints limiting the ability for credible provincial and federal sources to produce timely evidence syntheses that may be applicable for guiding provincial and local policy decisions. This was evident within agencies like Public Health Ontario (PHO), which would typically be involved with preparing evidence syntheses for the province. However, prior to the pandemic the agency was understaffed, and that created acute challenges when the pandemic hit:

*“The Ministry let their board be diminished. [.]. A lot of senior people left pre-COVID. They were losing a lot of talented and experienced individuals. So, by the time COVID hit there are not a lot of folks left quite frankly”*—*L1.*

#### 3.2.4 Combining credible evidence with local understanding

As evidence syntheses became more widely available, many participants reported challenges turning large amounts of evidence into actionable recommendations.

One approach adopted by many public health units involved incorporating the input from consultations with community stakeholders and using these documented descriptions of their lived experiences to inform decisions. Many officials at the provincial/territorial and local levels described engaging with business and municipal leaders and other relevant governmental agencies, both to hear concerns and to better understand potential effectiveness and risks of alternative actions. This process and its consequences are illustrated in the following quote from one provincial/territorial MOH:

*“We worked with industry groups, so we had a table that was pulled together by the Ministry of Industry and Trade, where we looked at what were the biggest risks of workplaces. One of the things set up was a workplace committee that included [the provincial/territorial worker safety and insurance authority], health authorities, senior representatives from key industries, and environmental health leaders; and we developed an order that required every workplace to have a COVID safety plan”—PT6.*

Another participant emphasized the importance of lived experience, particularly in environments where there was potentially elevated risk, as in long-term care:

*“One of the things that I am really proud of […] is the consultations that we did in the long-term care sector where we had so many town halls open to residents, families, staff, and employers to try to get, as much as possible, the perspectives from the people who were going to be the most impacted by the policies”—PT9.*

Beyond considering the quality and local relevance of available evidence, public health experts interviewed consistently discussed how the development of recommendations relied heavily on input and feedback from local communities, which further emphasizes earlier described themes surrounding the importance of locally relevant evidence in assessments of evidence quality. This was illustrated by one participant who noted:

*“Data and evidence never tell you what to do. [.] It always has to be put in the context of values, preferences, and judgments. […] My role is to bring the evidence, recommendations, and advice, and to work through it with the political leaders. What does that mean? How does that impact it? What can we do to support people who are more differentially affected by the orders?* Etc. *So it was a true partnership in developing the recommendations and the policies that we did through the pandemic, and that continues to today”—PT6.*

### 3.3 Challenges and tensions

Decision-makers struggled to navigate the extended evidence hierarchy, balancing different sources, assessing quality, and addressing the limits of models and lower-tier evidence. These challenges were compounded by the politicization of evidence and the urgency of making decisions under uncertainty.

#### 3.3.1 Integrating diverse evidence

Public health officials had to integrate varied and sometimes conflicting evidence while shaping policy recommendations during the pandemic. This meant working across the extended evidence hierarchy, from scientific studies and predictive models to lower-tier evidence and accounts of lived experience. Although some participants said that their role was focused was limited to public health effects, others attempted to incorporate evidence from other disciplines. As one participant noted, the challenge was making sense of multiple evidence sources:

*“It’s less about assessing the quality of each individual piece of evidence and more about how to integrate all of the evidence together. You’re getting so many different types of evidence about so many different facets of a policy decision. You’re getting epi evidence, evidence from community engagement, and evidence about the economic impacts. All of those are different types of evidence and all of them could be terrible in terms of quality. Especially at the beginning of the pandemic, we were getting lots of evidence, but all poor quality”*—*L2.*

Several public health decision-makers described using evidence from economics to assess the potential economic impact of public health policies. Most participants recognized the importance of considering these kinds of outcomes, as illustrated by one senior public health official:

*“Sometimes, [the policy tradeoffs are] mischaracterized as economics versus health. I think that’s inappropriate and contributes to a harmful narrative. […] Any of these decisions being made or interventions being taken had both some positive and some negative impact on people’s health. It really ignores the fact that our health is multi-dimensional. Considering the social determinants of health, the impact on employment, income, and social isolation, and all of the different [aspects of] healthy child development, were all a part of the decision-making process from the very beginning”—PT9.*

Several participants, particularly those in senior positions within provincial and territorial public health units, emphasized this sentiment. It alludes to the broader debate about how to weigh broader physical and mental health, child development, and economic considerations alongside direct health impacts in pandemic decision-making. However, integrating social science-based evidence, including economic models, into public health decision-making often came with challenges and sometimes skepticism. This skepticism was generally tied to the differences in methodological approaches used across different fields, where disciplines like economics typically rely on methodologies that would be viewed as lower-tier evidence in traditional evidence hierarchies. This is illustrated by one senior provincial/territorial MOH who noted:

*“The strength of the evidence is harder to demonstrate with social science. And so you get a lot more debate around what’s real and what’s true versus with the biomedical [evidence]”—PT9.*

Several others across local, provincial/territorial, and national units pointed out that this kind of evidence was outside the expertise of officials within public health units:

*“The economic and equity impacts [were] something that [our] infectious disease team would not do. They were overwhelmed already with what needed to be known [about health impacts]”—L5.*

Others also described this process of integrating evidence from different disciplines presented unique challenges, as illustrated by one national research director who noted that this required bringing diverse expertise and backgrounds together:

*“If you have an economist at every decision-making table, you should have a scientist at every decision-making table. It should be an ongoing conversation rather than an input. Science was a very big input during the pandemic, but there are usually also stakeholders involved”*—*N5.*

Integrating education-related evidence also presented difficulties. Decision-makers had to weigh the risks of school closures on children’s learning and well-being against the potential for schools to drive community transmission. Several subjects described consulting with educators and school officials as they worked to understand the impact on children. Some said that these efforts were limited by a lack of credible, real-time attendance and learning data. One participant described their experience working closely with their provincial Ministry of Education, though this collaborative experience was not necessarily universal across jurisdictions:

*“In terms of schools, our perspective throughout was last to close, first to open and, frustratingly, that was not what the Provincial Government did”—L1.*

*“The pediatric group gave their recommendations to the public health/education table that looked at things. Then it came to me. Then I had conversations with senior people in the Department of Education to either make decisions or bring forward a recommendation collectively between education and public health to the Premier’s office*”—*PT8.*

Some participants reflected on the fact that the decisions announced by policymakers were not necessarily consistent with public health recommendations. This was frequently the case when the evidence was less conclusive. These differences were frequently attributed to differences in underlying values, which may lead policymakers to be more sensitive to different priorities and objectives. This was illustrated by one provincial/territorial MOH who noted that:

*“Policy decisions are contextual, and inherently there are trade-offs with any decision and therefore values play into it”—PT9.*

The findings show how many public health officials integrated diverse evidence from across disciplines while accounting for local context, stakeholder values, and political constraints in shaping recommendations.

#### 3.3.2 The politicization of evidence

As the pandemic progressed and the psychosocial effects of shutdowns (e.g., isolation, learning loss, economic hardship) became more pronounced, the evidence was often seen as being increasingly politicized as different stakeholders selectively used and interpreted data to support their positions. Several participants, particularly those at national and provincial/territorial levels, who were more likely to interact more closely with political decision makers, noted that challenges arose when there was uncertainty in evidence. This uncertainty introduced opportunities for other factors to influence policy outcomes. One participant observed:

*“The policymakers really do value science and evidence. They just do not know what to do when the evidence is not clear and a decision still has to be made. At that point, they look at the media; they look at what the minister wants; they look at the economics; they look at public opinion; they look at who’s attacking them in the opposition”—N5.*

As discussed previously, many experts mentioned that differences in how evidence was used often stemmed from differences in stakeholder values. This was discussed by many participants in the context of evidence specifically since when there was uncertainty in the available evidence this was seen as an opportunity for these differing values to lead to conflicting conclusions on the appropriate policy decisions. Several participants noted that predictive models in particular came with challenges, including uncertainty, assumptions, communication, and political pressures. One participant described concerns about how model limitations were communicated and the risk of political interference:

*“One of the Cabinet Ministers was quoted as saying they did not trust the modeling, and they wanted to see if it happened first. Well, that’s not how you actually deal with a communicable disease. You do not wait for it to get bad. You have to actually anticipate and move earlier. But that did not happen, for the most part”—L1.*

This reflects the difficulty of using models in decision-making, especially when results seem uncertain or may contradict political priorities and counterfactual data is unavailable.

Evidence surrounding policies for economic or educational settings were two other areas that were perceived as particularly vulnerable to politicization. With respect to economic considerations, several subjects reported that they interacted with business lobbies and political leaders who pushed for reopening the economy. However, none of the participants in our study directly reflected on the emerging post-pandemic criticism that public health officials may have inadequately accounted for the policies’ broader educational, psychosocial, and economic impacts [e.g., (15–17)].

Many subjects expressed that their units were concerned with the impact of school closures, online learning, or stay-home orders on children. Several described how some decisions not to return to in-person education were politically motivated and went against public health recommendations. As a local public health official said, “*Our perspective throughout was [that schools should be] last to close, first to open and frustratingly, that was not what the Provincial Government did. (L1)*”

Multiple stakeholders described how studies on the impact of school closures on children’s learning, mental health, and social development were used by those advocating for a return to in-person schooling. Meanwhile, those arguing for continued caution cited evidence on transmission risks in schools, including for parents, teachers, and staff. These debates were often highly polarized, making it difficult to weigh trade-offs in a balanced way. Participants noted the challenge of integrating often opposed stakeholder perspectives and the degree to which things like school reopenings were contentious. For example, two participants working in national public health policy said:

*“We were trying to come up with recommendations about how to return to school safely, and when you should decide to add additional layers of interventions. And that was a situation where people just had extremely different views”—N3.*

*“People were very divided. Probably about half the people thought kids should stay home, and the other half thought kids should be going to school. And at that time the findings of our review were suggesting kids should be back in school. But what social media did with that was pretty interesting in terms of whoever disagreed with that really took us to town in terms of feeling like we were politically motivated”—N7.*

The politicization of evidence was complicated by concerns that communicating scientific uncertainty to the public could undermine trust in the evidence, while failure to do so could undermine trust in the system if the evidence changed. Several provincial/territorial MOH officials described how both the public and government struggled to interpret uncertainty in and the evolution of scientific evidence:

*“Quite a bit of the challenge was just trying to frame the uncertainty around evidence for leaders. I do not know for sure that it was low literacy [.] I think in some cases it was lack of familiarity” (PT12).*

*“I remember trying to be clear that this is what we know now and we are still learning. …I would be even clearer about explicitly saying, ‘The evidence will change. We will learn more. What I’m telling you today is what we know today, and we will know more tomorrow and next week’”—PT9.*

The pandemic highlighted that scientific evidence alone is often insufficient to resolve complex policy debates. Economic interests, political pressures, and competing values all shape how evidence is understood and applied.

In the following section, we describe how these themes have been integrated to develop a new conceptual framework for how evidence is used and assessed in public health decisions.

## 4 Toward a new framework of evidence assessment

Traditional evidence hierarchies rank scientific evidence based on methodological rigor, privileged study designs that minimize bias and maximize internal validity (1, 18). Typically, systematic reviews and meta-analyses sit atop these hierarchies, providing synthesized findings from multiple randomized controlled trials (RCTs), followed by individual RCTs, cohort studies, observational research, case studies, and expert opinion (19, 20). While this structured approach has significant value for guiding clinical practice and public health policy under normal conditions, our study reveals its limitations in rapidly evolving emergency contexts like the COVID-19 pandemic, where high-tier evidence often emerges too slowly to meet urgent decision-making demands.

In rapidly evolving information environments, officials cannot delay decisions until higher-tier scientific evidence emerges. Instead, they must make practical judgments based on available evidence, expert input, and broader considerations such as societal values, economic impacts, and political pressures (3). By integrating theories of crisis decision-making, evidence-based policy (EBP) models (2), and the sociology of scientific knowledge (SSK) (5, 6) into our analysis, we provide a multifaceted assessment of how public health leaders in Canada acquired, perceived, and implemented evidence during the pandemic. In doing so, we also identify lessons for improving evidence use and coordination in future public health emergencies, particularly regarding the importance of trust, transparency, and adaptability in decision-making.

Our findings demonstrate that rigid adherence to traditional hierarchies based solely on methodological rigor (systematic reviews, meta-analyses, and randomized controlled trials (RCTs)) proved impractical during urgent, rapidly evolving situations. As seen in past outbreaks like the 2009 H1N1 influenza pandemic, beyond reliance on established public health principles, public health decision-makers often looked to lower-tier evidence, including international and observational data, pre-prints, expert opinion, and anecdotal reports to inform decisions (21). Public health officials instead adopted a pragmatic, multi-dimensional approach that explicitly considered methodological rigor, source credibility, and applicability of the available evidence.

### 4.1 Evidence assessment during crisis: the methodology-credibility-applicability framework

Drawing on our empirical results, we propose a multi-dimensional framework for understanding the assessment and integration of evidence in crises: the Methodology-Credibility-Applicability (MCA) Framework. This is based on three key themes that emerged within the data, which are described in Section 3.

First, throughout the data, there was widespread consensus that evidence sources that were higher within the traditional evidence hierarchy were preferred, as illustrated by the findings in Sections 3.1 and 3.2. This has important implications for how evidence from different disciplines are integrated into decision making, as meta-analyses and other methodologies that are higher within the evidence hierarchy are less common in some relevant social sciences.

Second, experts were highly sensitive to the credibility of the source, regardless of where the methodology fell within the traditional evidence hierarchy. Practitioners were critical of all evidence they were faced with and consistently sought out evidence that was deemed more credible, particularly during a crisis, where the methodology alone was a less reliable indicator of the quality of a given information source. This is based on the findings presented in Sections 3.2.2–3.2.4, with additional challenges related to assessments of evidence credibility discussed in Section 3.3.

Third, decision makers prioritized data and evidence that was locally relevant and applicable to their own decisions, as illustrated by the findings presented in Section 3.2. As demonstrated by the findings presented in Section 3.3.1, this localization of evidence was essential during an emergency situation when each location was experiencing different challenges and there were no systematic reviews that could help decision makers navigate which aspects of studies from other environments likely applied to them.

Together, these findings suggest that, rather than a single linear hierarchy, evidence quality and usability during emergencies should be simultaneously evaluated across three key dimensions:

*Methodology*: This dimension reflects the traditional evidence hierarchy, expanded to explicitly include predictive modeling, placing it below systematic reviews and RCTs but above observational studies and expert opinion. The relative position reflects how they were widely treated by public health leaders, who perceived the models as a structured approach founded on first principles, incorporating the most relevant observational data, and providing insights tailored to specific scenarios (11).

*Credibility*: Assesses the study design and the reputation, expertise, independence, and reliability of evidence producers, publishers, or endorsers, such as respected institutions (WHO, CDC) or recognized scientific communities (2). Pre-prints, for instance, though traditionally seen as less reliable, became critical and reliable sources when authored by reputable institutions or experts during the pandemic, particularly when other more conventional sources were unavailable.

*Applicability*: Considers how relevant, context-specific, and immediately actionable evidence is to decisions within the local public health context. Even methodologically weaker local data may be prioritized over robust international meta-analyses if the local applicability is higher (22). Participants in our study, for example, stressed the importance of local surveillance data, which became more influential over time as decision-makers sought evidence tailored to their specific epidemiological and community contexts. This reflects the public health axiom that “good public health is local” (14) and that equity-driven policy creation is often also the most effective way to achieve public health at a population level.

Our analysis showed that decision-makers utilized these three dimensions simultaneously, selecting and integrating evidence based on a balance between timeliness and comprehensiveness. This flexible approach allowed officials to address urgent policy decisions even when high-tier evidence was lacking.

Policymaking during a crisis involves more than identifying a single, optimal piece of evidence to guide action. Instead, decision-makers must weigh and integrate multiple, often imperfect, sources of evidence alongside foundational scientific knowledge and established public health principles, collectively referred to as first principles, as discussed in Section 3.1. Our findings indicate that public health officials systematically assessed the quality, credibility, and relevance of available evidence before combining these assessments with first principles and their professional expertise to inform policy decisions.

**Figure 1:**
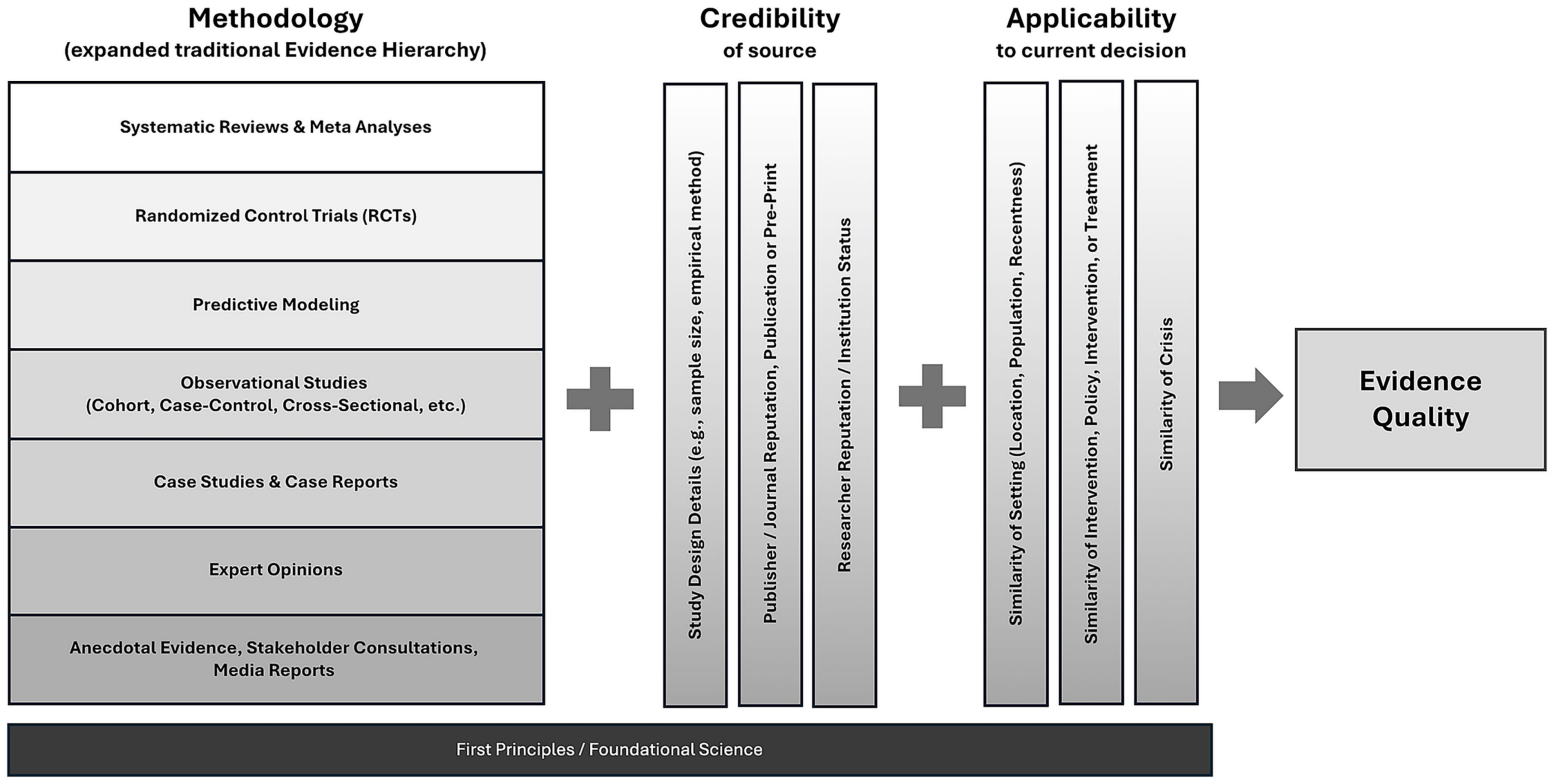
MCA evidence quality framework.

First principles played a critical role throughout the pandemic, particularly when high-quality, context-specific empirical evidence was lacking. In such scenarios, decision-makers relied heavily on deductive reasoning grounded in fundamental understandings of disease transmission and public health practice (23). The need for rapid, decisive action under significant uncertainty and high stakes further reinforced this reliance on first principles, highlighting the limitations of traditional evidence hierarchies during acute crises (2, 21). At the same time, first principles offer practitioners a constant point of reference as they consider the methodology, credibility, and applicability of evidence. This is reflected in Figure 1, where first principles are included as a foundational consideration across the MCA. When practitioners faced a lack of evidence or evidence based on methodologies low on the traditional evidence hierarchy, not from credible sources, or not applicable to the context they were making decisions for, they could refer to first principles as a guide for making decisions. This flexible and integrated approach allowed public health leaders to act swiftly and effectively despite significant knowledge gaps and evolving evidence landscapes.

Table 2 reviews the Methodology, Credibility, and Applicability for several of the lower-tier or non-traditional evidence sources used by public health decision makers during COVID-19. The selected examples are consistent with those mentioned in the interviews and in other sources. While the interviews did not explicitly highlight the use of social media and mobility data, we include them in the summary table based on the increasing recognition as a useful source of real-time, local data during crisis events [e.g., (24, 25)].

**Table 2:** MCA framework in practice: non-traditional evidence during COVID-19.

Evidence type	Methodology	Credibility	Applicability
Predictive modeling	Mid-tier—above observational studies, below RCTs/quasi-experiments	Depends on model developers (e.g., PHAC vs. *ad hoc* non-governmental roundtables)	High—scenarios tailored to local transmission data
Local wastewater surveillance	Low-tier—environmental surveillance	Higher when conducted in partnership with trusted labs/universities	Very high—directly reflects local viral trends
Stakeholder consultations	Low-tier—anecdotal evidence and expert opinion	Depends on stakeholder participants and who is convening; often seen as politically motivated	Very high—captures community-specific concerns and values
Preprint studies	Depends on methodology of the study	Substantially lower than peer-reviewed studies; depends on reputation of author/institution	Medium—can include local case studies but not always context-specific
Mobility data	Low- to mid-tier—depending on analytics; proxy observational data	Data credibility high when from credible data source; analytics quality may be questioned	High—reflects local compliance and movement patterns
Social media analytics	Low- to mid-tier—depending on analytics; proxy “sentiment” observational data	Depends—more credible when conducted by recognized research centers	Medium–high—can be segmented by region or demographic
International observational case reports	Low-tier—observational studies	Higher if from WHO, CDC, UK’s NHS etc.; lower if ad hoc country reports	Low—context may differ substantially
Science advisory groups and expert panels	Depends—mix between low-tier expert opinion and synthesis of mostly low- and mid-tier evidence	Typically high when convened by federal/provincial scientific advisory tables with vetted members	Medium—recommendations may need local adaptation
Economic modeling	Mid-tier—similar status to predictive models (structured, data-driven projections)	Depends on modelers’ reputation (e.g., central bank vs. private consultants)	Medium—can be localized by region but often uses national aggregates

Our MCA framework builds on and extends existing approaches such as the GRADE system (Grading of Recommendations, Assessment, Development, and Evaluation) (20). While GRADE offers a structured method for assessing methodological quality, it treats factors like applicability and source credibility as secondary considerations. In contrast, the MCA framework elevates applicability and credibility to equal footing with methodology, better reflecting the realities of public health crises where decision-makers must act on incomplete, rapidly evolving evidence from diverse sources. MCA complements GRADE by providing a more flexible, context-sensitive approach suited to emergency decision-making.

Although the evidence used to conceptualize this new framework stems from interviews with Canadian public health experts, the interviews with these experts frequently included discussion of factors that are not specific to Canada. Indeed, the diversity of public health governance systems across provinces and territories in Canada suggest that this framework can be flexibly applied to contexts with different governance systems. This could include settings where there are multiple jurisdictional authorities (e.g., federal, state and local agencies, like the United States) as well as more centralized governance structures such as the United Kingdom. The MCA frameworks illustrates how to assess the quality of evidence sources that inform a wide range of common public health decisions—such as best practices for contact tracing—while also assessing the applicability of the evidence being considered (14). These criteria are relevant to diverse public health and decisions environments. However, as Table 2 illustrates, the weight of each criteria in the MCA framework will be assessed differently for different jurisdictional levels or types of decisions.

### 4.2 Considering an expanded set of evidence

The MCA framework emphasizes that evidence-informed decision-making in public health crises involves more than evaluating individual studies independently; it requires a broader consideration of multiple evidence types simultaneously. Our findings illustrate how predictive models, emerging local data, and evidence synthesis became critical resources throughout the COVID-19 pandemic. Decision-makers frequently had to assess these varied and evolving sources of evidence, highlighting the importance of an explicit, systematic approach to guide integration and application in crisis contexts. This is especially true as technology increases capacity for data collection and predictive analysis. The following sections discuss the implications of relying on an expanded set of evidence types, including predictive models and localized data, as revealed by our findings.

#### 4.2.1 Predictive models as essential evidence

The expanded methodological hierarchy under the MCA framework explicitly positions predictive models alongside empirical studies. During the COVID-19 pandemic, these models became essential for integrating and analyzing rapidly changing data, allowing public health officials to visualize scenarios and outcomes. Despite their utility, participants expressed concerns about inherent uncertainties, transparency of assumptions, and potential political misuse. Enhancing predictive models’ credibility requires clear communication, transparent development, and independent validation (26). The most influential models during the pandemic were those endorsed by credible modeling groups or advisory bodies.

Our analysis also identified a gap in integrating rigorous economic modeling into public health decision-making, despite recognizing the importance of economic concerns. Strengthening interdisciplinary collaboration between economists and public health officials could bridge this gap, ensuring broader awareness and trust in comprehensive modeling analyses (27, 28, 30).

#### 4.2.2 Local data and emerging evidence sources

Participants emphasized the increasing importance of localized evidence sources, including wastewater surveillance and community-level epidemiological data, despite these sources traditionally ranking lower methodologically. These types of evidence, however, often proved crucial due to their high applicability and timeliness in local contexts. Recognizing such data’s value underscores the need to incorporate emerging evidence sources explicitly into frameworks guiding public health decision-making, and to build partnerships where it is impractical or impossible to house that capacity within public health organizations themselves.

#### 4.2.3 Strengthening evidence synthesis and knowledge translation

Participants highly valued synthesis efforts (e.g., provincial scientific advisory committees) in managing extensive, evolving evidence bases. However, variability in capacity across jurisdictions highlighted the necessity of ongoing investment in rapid and credible evidence-synthesis infrastructures. Similarly, subjective perceptions of limited applicability hampered the application of evidence in areas where custom products were not available. Integrating lived experiences and qualitative insights from stakeholders into these syntheses aligns with participatory governance principles, acknowledging evidence as encompassing scientific data, community values, and experiential knowledge (6, 29). In general, efforts during non-crisis times to develop flexible and efficient mechanisms to maximize capacity for evidence generation and synthesis could help reduce duplication of effort and ease pressure on strained human health resources during future pandemics.

#### 4.2.4 Establishing systematic approaches beyond methodology

While traditional evidence hierarchies provide well-established frameworks for evaluating evidence based on methodological rigor, our findings highlight a notable absence of systematic standards for assessing the credibility and applicability dimensions. Public health officials routinely considered author reputation, institutional credibility, publisher quality, and the reliability of preprints during the COVID-19 pandemic, but these assessments were often informal and lacked consistent criteria. There is also limited guidance on systematically determining the appropriateness of research design for specific public health contexts or the direct applicability of study findings to local decision-making contexts (22, 37).

Developing formalized criteria and systematic hierarchies for credibility and applicability could enhance decision-making rigor during future crises by reducing subjectivity and improving transparency in evidence selection processes. Explicitly codified standards for evaluating author expertise, institutional independence, and context-specific applicability can complement existing methodological hierarchies, creating a more robust and comprehensive approach to evidence assessment (2, 23). Such efforts would contribute significantly to strengthening evidence-informed policy and decision-making within public health, particularly in high-stakes and uncertain emergency contexts.

#### 4.2.5 Limitations

The development of the MCA framework builds on detailed interviews with Canadian public health experts from a diverse set of agencies and senior roles. Although many of the insights from the Canadian context likely carry over to crisis decision making in other countries and contexts, additional research is needed to validate the framework in other settings or for other forms of crisis. Similarly, although our subjects sometimes provided insight into how politicians or other stakeholders interpreted and engaged with evidence, any such perspectives are interpreted through the lens of the public health officials in our study. Additional research is needed to understand whether and how the MCA framework is relevant to other decision makers outside public health, as well as the community stakeholder groups that PH agencies engaged with (e.g., frontline workers, business associations, community groups). Other groups may have different approaches to evaluating and using evidence, and different perspectives on the inherent challenges faced during COVID-19. For example, Brubacher et al. (25) interviewed a broader set of community members and decision makers, reporting a general lack of clarity around what evidence influenced policy and how it did so. Furthermore, although the current study has compared the validity of the MCA framework to other evidence frameworks in the literature [e.g., (20)], it does not formally validate the framework with participants directly. This provides an opportunity for further research to achieve this type of triangulation.

Methodologically, there are other important possible limitations. Despite the research team establishing a rapport with interview participants that seemingly fostered honest discussion, it is possible that more senior individuals in the sample may have retrospective cognitive biases (e.g., cognitive dissonance reduction, hindsight bias, recall bias, motivated reasoning) that led them to unintentionally misremember or misstate certain aspects of their experiences during the COVID-19 crisis. To mitigate these unavoidable limitations, the research team compared themes across individuals from different regions, jurisdictions, and seniority levels, probed for specific examples, and reviewed participant-provided and independent documents written during the height of the pandemic to confirm participant reports.

## 5 Discussion

This study provides two substantial contributions to the literature on evidence-informed decision-making during public health crises.

First, it offers unique empirical insights from extensive qualitative interviews with senior public health decision-makers across Canada, capturing their experiences navigating complex and rapidly evolving evidence landscapes during the COVID-19 pandemic. These firsthand accounts reveal the pragmatic, multidimensional, and context-sensitive approaches officials used to assess and integrate diverse forms of evidence, including predictive models, local surveillance data, preprints, stakeholder input, and interdisciplinary insights, all while balancing methodological rigor with practical policy demands and political realities.

Second, building upon these empirical insights, we introduce the Methodology-Credibility-Applicability (MCA) Framework, a novel, structured yet flexible approach for understanding evidence assessment during emergencies. Unlike traditional hierarchical models emphasizing methodological rigor alone, the MCA Framework equally incorporates source credibility and local applicability dimensions, reflecting the practical realities public health officials face during crises. Our findings demonstrate that timely, credible, and contextually relevant evidence often proves more influential and actionable than more robust yet less applicable international studies.

Beyond the MCA Framework, our study underscores the urgent need to strengthen infrastructure supporting rapid evidence synthesis and interdisciplinary collaboration. Enhancing transparency and clear communication regarding predictive modeling, alongside systematic assessment of the credibility and applicability of evidence sources, can bolster public trust and improve decision-making effectiveness. These implications extend beyond the Canadian context, offering valuable lessons for policymakers, practitioners, and researchers globally as they prepare for future public health emergencies. Given Canada’s federalist government system and public health structure that gives significant independence across provinces, some of the findings regarding duplication of effort, coordination, and evidence applicability across jurisdictions may be specific to Canada.

Broader practical implications for public health practice include the development of flexible guidelines explicitly integrating diverse evidence types such as lower-tier evidence, predictive models, stakeholder experiences, and qualitative insights. Investments in robust, equitable, rapid-response infrastructure for evidence synthesis are critical, particularly in resource-constrained jurisdictions. Enhancing interdisciplinary collaboration, especially across epidemiology, economics, education, and community stakeholders, is essential for fostering comprehensive, trusted decision-making processes. Additionally, targeted training for public health professionals on effectively communicating uncertainties and evidence limitations can reinforce public confidence and enhance decision-making transparency during crises.

However, the insights that build into the MCA framework, including the importance of first principles, the reliance on different evidence types, and concerns about credibility and applicability, not just methodology, remained relatively similar across all jurisdictions in our study. There is also no aspect of the MCA framework that is specific to the Canadian public health structure. These factors suggest that the MCA framework can guide the assessment of evidence in other settings as well, including those with a blend of federal and provincial or state control (such as the United States), as well as those that are more centralized (such as the United Kingdom). This is likely true given that even when public health is governed by one central agency, there will still be some need to contextualize public health recommendations at a local level (14). Future research should empirically evaluate the MCA framework’s reliability and effectiveness across various crisis and urgent-response contexts, exploring additional categories within the Credibility and Applicability dimensions and refining the placement of predictive models within evidence hierarchies. This could include having public health experts at different jurisdictional or seniority levels judge various evidence sources according to the criteria in the MCA framework, then comparing this to how each expert would choose to use the different sources in hypothetical scenarios.

Developing standardized assessment protocols, advancing interdisciplinary collaboration practices, and improving strategies for uncertainty communication are critical avenues for strengthening public health preparedness and responsiveness in future emergencies. Furthermore, even though the MCA framework has been developed in the context of a public health crisis, the evidence dimensions of Credibility and Applicability may be relevant for assessing evidence more generally, recognizing that not all studies that apply similar methods are equally reliable or relevant.

## Data Availability

The original contributions presented in the study are included in the article/supplementary material, further inquiries can be directed to the corresponding author.
